# The Impact of General Anesthetics on Postoperative Delirium: A Narrative Review Based on Clinical Randomized Controlled Trials from the Last Five Years

**DOI:** 10.3390/geriatrics11030070

**Published:** 2026-06-12

**Authors:** Jia-Ni Wu, Jia-Huan Xu, Jia-Yi Ge, Bo-Ran Deng, Xing-Jun Liu

**Affiliations:** School of Pharmacy, Nantong University, Nantong 226019, China; 18068396686@163.com (J.-N.W.); 17805091070@163.com (J.-H.X.); 13275103662@163.com (J.-Y.G.); boratteng007@gmail.com (B.-R.D.)

**Keywords:** postoperative delirium, anesthetics, dexmedetomidine, benzodiazepines, opioids

## Abstract

Postoperative delirium (POD) is an acute, reversible neurocognitive disorder characterized by confusion and altered consciousness. With the improvement in research methodologies and the introduction of innovative clinical drugs in recent years, a growing number of randomized controlled trials have been conducted. This article aims to conduct a comprehensive review of the efficacy of general anesthetics—including propofol, ciprofol, sevoflurane, ketamine, esketamine, dexmedetomidine, benzodiazepines, opioids, and lidocaine—in preventing and managing POD, based on randomized controlled trials published in the past five years. Propofol has advantages in preventing POD in pediatric patients. However, its efficacy compared with inhalational anesthetics still requires individualized evaluation in elderly patients. The novel drugs ciprofol and remimazolam exhibit favorable safety profiles and do not increase the risk of POD. The efficacy of dexmedetomidine shows variability across patient populations and surgical types. In addition, specific opioid drugs and lidocaine also demonstrate preventive potential when administered in a standardized manner.

## 1. Introduction

Postoperative delirium (POD) is an acute and reversible dysfunction of the central nervous system that typically manifests within hours to days following surgery. It is primarily characterized by alterations in consciousness, cognitive disturbances, and behavioral abnormalities. Key risk factors include advanced age, cognitive impairment, frailty, cardiovascular disease, renal impairment, psychiatric disorders, malnutrition, chronic alcoholism, and sensory deficits (e.g., visual and hearing impairment). Notably, the perioperative use of medications such as benzodiazepines, anticholinergics, dihydropyridine calcium channel blockers, and opioids, along with dehydration and psychological stress, may also precipitate POD [1].

The global incidence of POD ranges from 10% to 60% across elective and emergency surgical procedures [2]. POD frequently results in adverse outcomes, such as falls, bed-related injuries, delayed recovery, increased healthcare costs, prolonged hospital stays, heightened mortality rates, and delayed cognitive restoration. It imposes a substantial burden on patients’ families and healthcare resources [3]. Therefore, it is imperative for clinicians to swiftly identify risk factors associated with POD, mitigate precipitating factors, and implement effective preventive and therapeutic strategies.

Currently, the global prevalence of general anesthesia in low- and middle-income countries exceeds 80%. In high-income nations, while the proportion of general anesthesia is lower, it remains the predominant method employed [4]. This trend can be attributed to various factors. Certain regions face shortages of qualified anesthesiologists and inadequate equipment, alongside insufficient promotion of regional anesthesia techniques, leading to a preference for general anesthesia. For the vast majority of surgical procedures in fields such as cardiac, thoracic, otolaryngology, neurosurgery, and various laparoscopic interventions, general anesthesia is regarded as the optimal approach.

Furthermore, the growing demand among patients for comfort in medical care has significantly fueled the rise in general anesthesia utilization. Patients undergoing general anesthesia are exposed to a range of anesthetic agents, including traditional medications such as propofol, sevoflurane, isoflurane, and ketamine, as well as newer agents introduced in recent years, such as ciprofol, remimazolam, and esketamine. However, the impact of various anesthetic agents on the occurrence of POD continues to be a subject of significant debate.

With advancements in research methodologies and the introduction of innovative clinical drugs, the number of clinical trials investigating the effects of general anesthetics on POD has surged. We systematically searched for randomized controlled trials (RCTs) conducted over the past five years (July 2020 to July 2025) that investigated the association between anesthetic agents and POD. This review synthesizes the findings of these studies to deepen the understanding of perioperative POD prevention and management (Table 1).

Before further discussion, it is necessary to clarify the definitions of different age groups used in this article: children/pediatric patients refer to individuals aged <18 years; adults refer to individuals aged ≥18 years and <65 years; and older adults refer to individuals aged ≥65 years. In this article, the terms “children” and “pediatric patients”, “adults” and “non-elderly adult patients” are used interchangeably, all following the above definitions.

In addition, emergence delirium (ED) and emergence agitation (EA) are early manifestations of POD during the anesthetic emergence period, particularly prevalent in children, characterized by restlessness, crying, and disorientation, usually occurring within 30–60 min after awakening. Although these conditions are not completely equivalent to POD, they have a strong correlation with POD and are therefore discussed collectively in this review.

## 2. Literature Search and Screening Methods

We conducted a systematic search of three databases: Web of Science Core Collection, Scopus, and Google Scholar, covering the time period from July 2020 to July 2025. The search strategy combined Medical Subject Headings and free-text terms: (“postoperative delirium” OR “emergence delirium” OR “emergence agitation”) AND (“general anesthetics” OR “intravenous anesthetics” OR “inhalational anesthetics”) AND (RCT).

Inclusion criteria: (1) Randomized controlled trials; (2) Study participants were patients undergoing general anesthesia; (3) The intervention was any type of general anesthetic agent; (4) Outcome measures included POD or ED/EA.

**Figure 1:**
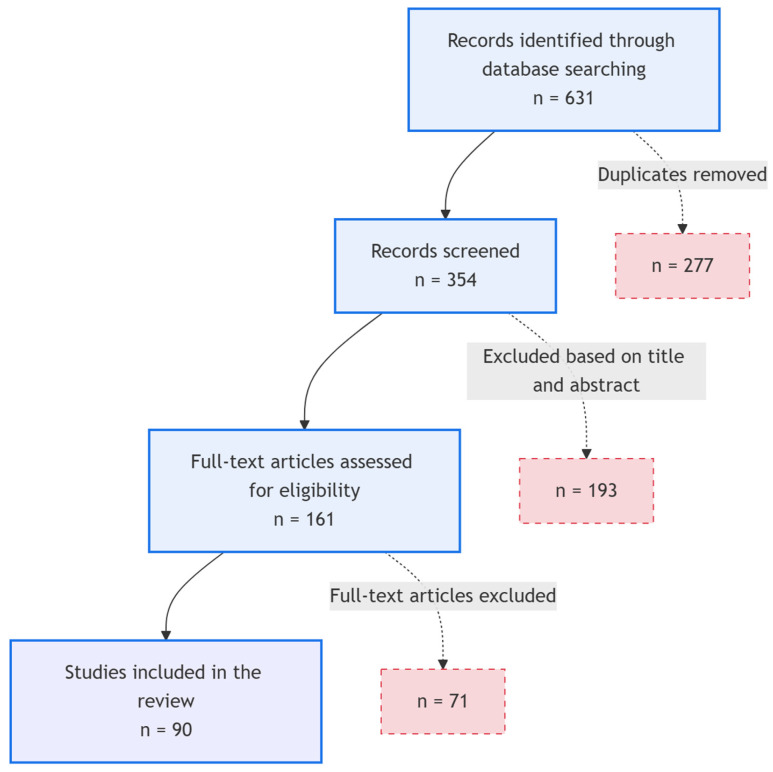
Flow Chart of Literature Retrieval and Screening.

Exclusion criteria: (1) Non-randomized controlled trials (e.g., observational studies, case reports, reviews); (2) Studies that did not clearly report the diagnostic criteria for POD; (3) Duplicate publications or studies with incomplete data. Two authors independently screened titles, abstracts, and full texts. Disagreements were resolved through discussion or arbitration by a third author. A total of 90 RCTs were finally included. The study screening process is shown in Figure 1.

## 3. Pathophysiological Mechanisms of POD

The development of POD is multifactorial. The currently recognized core mechanisms include: (1) Neuroinflammatory response: Surgical trauma and anesthetic agents can activate microglia, releasing inflammatory cytokines such as interleukin-6 and tumor necrosis factor-α, leading to an inflammatory cascade in the central nervous system [5]. (2) Cholinergic dysfunction: Elderly patients have a physiological decline in brain acetylcholine levels, and anesthetic agents can further inhibit cholinergic neurotransmission, inducing cognitive dysfunction [6]. (3) Electroencephalographic suppression: Excessive anesthetic depth can lead to reduced power of alpha and beta waves on electroencephalography, which is closely associated with the development of POD [7]. (4) Blood–brain barrier disruption: Surgical stress and inflammatory responses can increase blood–brain barrier permeability, allowing peripheral inflammatory cytokines to enter the central nervous system [8]. (5) Sleep–wake cycle disturbance: Anesthetic agents can interfere with melatonin secretion and circadian rhythm, resulting in disruption of sleep architecture and increased risk of POD [9]. (6) Age-related susceptibility: Elderly patients have decreased cerebral reserve capacity and are more susceptible to the neurotoxicity of anesthetic agents [10].

## 4. Propofol

Propofol is the most widely used intravenous anesthetic in clinical practice. It enhances inhibitory activity by activating gamma-aminobutyric acid type A (GABAA) receptors and glycine-gated ion channels. Since its introduction several decades ago, many studies have emerged investigating various aspects of propofol. However, in recent years, RCTs on the impact of propofol on POD have been extremely limited, with only one clinical trial that focuses exclusively on pediatric patients and does not involve adult or older adult populations.

ED can be considered a subtype or early manifestation of POD, particularly when delirium presents as a hyperactive variant. In a double-blind trial, researchers administered propofol to 160 preschool children (aged 2–5 y) undergoing laparoscopic inguinal hernia repair under sevoflurane anesthesia at different time points. They used the PAED Scale combined with the Watcha Scale to assess whether ED occurred in the children. The findings revealed that the incidence of ED in the group receiving a continuous infusion of propofol at 1 mg/kg over three minutes was significantly lower than that in the other groups (*p* < 0.05) [11].

Recent studies have shown that propofol is relatively effective in preventing POD in pediatric patients. However, since the study was single-center, there may be a risk of selection bias. Given that propofol is widely used and carries the dual risks of injection pain and significant circulatory suppression, its comparative effectiveness against other anesthetics remains to be clarified.

## 5. Ciprofol

Ciprofol is a novel GABAA receptor agonist that bears structural similarities to propofol, offering several advantages, including minimal effects on the respiratory and circulatory systems, reduced injection pain, rapid onset, and quick recovery. However, at present, there is only one small-sample RCT examining the impact of ciprofol on POD. The trial included 84 elderly patients undergoing thoracoscopic lung cancer surgery and monitored the incidence of POD within the first three postoperative days. The findings indicated no significant differences between ciprofol and propofol. The incidence of POD in the ciprofol group and propofol group was 7.1% and 16.7%, respectively, risk ratio [RR] = 0.37, 95% confidence interval [CI] = 0.07–2.03, risk difference [RD] = −9.6%, 95% confidence interval = −23.3–4.1%, *p* = 0.178. But the Confusion Assessment Method (CAM) scores were notably lower [12], [13 (12, 15) vs. 15 (14, 17); 12 (11, 13) vs. 14 (13, 16); 12 (11, 12) vs. 13 (12, 14), *p* < 0.05]. Furthermore, a trial comparing recovery from anesthesia using ciprofol and remimazolam did not report any cases of POD [13].

At present, the application of ciprofol is limited to the induction, and relevant clinical evidence remains constrained by factors such as single-center design, relatively small sample sizes, and narrow outcome dimensions. A previous retrospective study found that this drug resulted in a low incidence of POD in 6-year-old children, but no firm conclusion can be drawn for elderly patients.

## 6. Sevoflurane

Sevoflurane is a fluorinated methyl isopropyl ether inhalational anesthetic, distinguished by its low blood–gas partition coefficient of 0.65, which facilitates rapid induction and recovery, and trails only behind desflurane and nitrous oxide in this regard. Owing to its favorable safety profile and relatively low cost, sevoflurane has become the most widely used volatile anesthetic in clinical practice.

General anesthesia and surgical procedures can increase levels of neuroinjury biomarkers, with these increases being linked to POD. Previous studies demonstrated that in patients undergoing extracorporeal shock wave lithotripsy, the administration of sevoflurane resulted in a significant rise in neuroinjury biomarkers, including total tau protein, neurofilament light chain protein, and phosphorylated tau protein at threonine 181. These markers peaked one hour postoperatively but returned to baseline levels after five hours [14]. Like propofol, sevoflurane has been the subject of few RCTs on POD in recent years. A single-center, prospective RCT compared the effects of sevoflurane and desflurane in elderly patients undergoing general anesthesia for total knee arthroplasty [15]. Researchers collected salivary melatonin at 14 time points, ranging from 1 day before surgery to 3 days after surgery. The results showed that there was no significant difference in salivary melatonin concentration between the two groups of patients at all time points. Since melatonin is closely related to postoperative circadian rhythm, and circadian rhythm disturbance is one of the high-risk factors for POD, this result may indirectly indicate that there is no difference in the impact of these two inhalational anesthetics on POD in such patients. In another study with a sample of only 30 patients, researchers compared the intraoperative electroencephalogram (EEG) results between patients receiving sevoflurane and those receiving desflurane [16]. For the diagnosis of POD, the CAM was used as the primary tool, supplemented by the Intensive Care Delirium Screening Checklist (ICDSC) to conduct a comprehensive assessment. They found that among patients who received sevoflurane, those with POD had significantly lower EEG spectral power in the 7.5–31.5 Hz band, reduced coherence in the 8.9–23.8 Hz band, and lower pairwise phase consistency in the α band compared to non-delirium patients (*p* < 0.005). Additionally, the area under the curve (AUC) values of α band power, β band power, and coherence in the sevoflurane group (0.72–0.88, *p* < 0.005) were significantly higher than those in the desflurane group (0.58–0.68, *p* > 0.043).

In summary, recent studies on sevoflurane and POD have mainly reported indirect associations, leading to a decrease in the quality of evidence. Probably because sevoflurane has been used clinically for many years and relevant research is relatively sufficient, researchers have paid more attention to exploring measures to reduce the incidence of POD under sevoflurane anesthesia. Considering the widespread use of sevoflurane, it may be beneficial for clinicians to focus on intervention measures that can reduce POD.

## 7. Inhalational Anesthetics vs. Intravenous Anesthetics

Inhalational anesthesia (IA) and total intravenous anesthesia (TIVA) represent the two principal techniques employed to maintain depth of anesthesia in contemporary general anesthesia. Almost all patients undergoing general anesthesia will be administered one of these methods or a combination of both. Moreover, with the continual introduction of new anesthetic agents, a plethora of comparative studies have emerged in recent years. This chapter primarily discusses RCTs designed to compare IA and TIVA.

A single-blind, prospective RCT found that, in pediatric patients undergoing tonsillectomy (with or without adenoidectomy), a propofol-based induction regimen was superior to sevoflurane in reducing the incidence of ED [17]. The PAED scores at 5, 10, and 30 min after surgery were 12.2 ± 4.2 vs. 9.1 ± 4.0 ( *p* = 0.003), 8.0 ± 2.6 vs. 5.1 ± 2.3 ( *p* < 0.001), and 5.1 ± 2.8 vs. 2.5 ± 1.8 ( *p* < 0.001), respectively. Researchers who compared the application of these two agents during the maintenance phase of anesthesia discovered a difference in the occurrence of ED within the first minute postoperatively—those receiving propofol exhibited a lower incidence, and children who experienced ED were more likely to demonstrate adverse behavioral changes afterward [18]. Taken together, from the perspective of POD incidence in pediatric patients, intravenous anesthesia demonstrates significant advantages in both the anesthetic induction phase and maintenance phase.

A study published in 2023 highlighted that for elderly patients aged 65 and above undergoing orthopedic surgery, there was no discernible difference in POD incidence between isoflurane inhalation anesthesia and propofol total intravenous anesthesia (14% vs. 13%, *p* = 0.84) [19]. Notably, this study analyzed data collected from November 2008 to December 2012, and led to a decade-long delay before the results were disseminated. Shin et al. [20] conducted a similar trial using desflurane and propofol and obtained similar results. In contrast, another study conducted across 14 major hospitals in China used the Confusion Assessment Method for the Intensive Care Unit (CAM-ICU) to assess 1228 elderly patients undergoing extensive cancer surgeries. The study found that the incidence of POD after propofol anesthesia was one-third lower than after sevoflurane anesthesia (8.4% vs. 14.4%), with a relative risk of 0.68 (95% CI: 0.48–0.95, *p* = 0.023); the adjusted RR was 0.59 (95% CI: 0.39–0.90, *p* = 0.014) [21]. Therefore, in the elderly population, the effects of inhalational anesthesia and intravenous anesthesia on POD may vary depending on patient status, specific medications, and surgical type.

Several recent comparative studies have encompassed adult patients, including the elderly, and these findings warrant discussion. Researchers compared the effects of maintenance anesthesia with volatile anesthetics (sevoflurane/desflurane) versus propofol-based TIVA on POD in 684 adult patients undergoing cardiac valve surgery under extracorporeal circulation or combined valvular and coronary artery bypass grafting [22]. Using the CAM-ICU to evaluate POD, they found no statistically significant differences between the two groups in either the 7-day incidence or the duration of POD (18.7% vs. 22.4%, RR = 0.80, 95% CI: 0.55–1.16, *p* = 0.231). However, this trial assessed POD only once daily, which may have underestimated delirium incidence. Additionally, the researchers did not restrict the use of propofol during the patients’ ICU stay. However, a small-sample clinical study indicated that propofol-based TIVA was associated with a lower incidence of POD in patients undergoing coronary artery bypass grafting compared to sevoflurane [23]. Another small-sample, prospective, randomized single-blind study showed that in adults (aged 18–80) undergoing elective coronary artery bypass grafting (CABG) under extracorporeal circulation, propofol-based TIVA significantly reduced the incidence of POD compared with sevoflurane-based IA (8.8% vs. 34.2%, *p* = 0.027). This effect was more prominent in patients aged 65 and above (14.2% vs. 63.6%, *p* = 0.03). However, the trial was significantly inadequate in controlling confounding factors, such as the use of postoperative dexmedetomidine.

To summarize, pediatric patients may be more suitable for TIVA. However, for elderly patients, it is necessary to formulate individualized, appropriate anesthetic plans based on factors such as surgical type and patient physical status. In addition, existing studies are mainly limited to the use of a narrow range of anesthetic agents; future research can explore the efficacy of various inhalational or intravenous anesthetics. Furthermore, investigating the comparative effectiveness of combined inhalational and intravenous anesthesia versus single anesthetic techniques may also be of certain value.

## 8. Ketamine

Ketamine is an N-methyl-D-aspartate (NMDA) receptor antagonist and the sole intravenous anesthetic that combines potent sedative and analgesic properties. However, its well-documented hallucinogenic effects have restricted its clinical applications.

Previous studies have shown that in pediatric patients undergoing tonsillectomy, the addition of 2 mg/kg ketamine via nasal drops to a 2 μg/kg dose of dexmedetomidine did not increase the incidence of POD (assessed by the PAED scale) [24]. Additionally, other researchers compared three different administration regimens in children undergoing tonsillectomy [25]. They used a combination of propofol and ketamine, with the specific dose pairs being: 1 mg/kg propofol and 1 mg/kg ketamine, 1.5 mg/kg propofol and 0.75 mg/kg ketamine, 2 mg/kg propofol and 0.66 mg/kg ketamine, and 3 mg/kg propofol and 0 mg/kg ketamine. The researchers evaluated the status of ED using the Watcha scale. The results showed that continuous infusion of propofol and ketamine at a 3:1 ratio effectively reduced ED incidence (*p* < 0.001) and provided sufficient postoperative analgesia.

It has also been found that in elderly patients undergoing emergency surgery for intestinal obstruction, ketofol (ketamine:propofol = 1:4) infused intraoperatively and within 2 h postoperatively had equivalent efficacy in reducing the incidence of POD compared with dexmedetomidine (assessed by CAM-ICU). Additionally, both were significantly superior to placebo, with the incidences being 5%, 2.5%, and 20%, respectively [26]. Researchers found that in patients aged 65 and above, administering ketamine at a rate of 1 mg/kg/h during cardiopulmonary bypass led to fewer incidences of POD within 24 h, compared with propofol administered at a dose range of 1.5–6 mg/kg/h [27]. However, the researchers of this trial stated that due to constraints in the trial conditions (small sample size and failure to rule out type II error), the preventive efficacy of ketamine against POD remains unclear. A study by Cerhan et al. [28] found that in elderly high-risk patients undergoing cardiac surgery, there was no significant difference in the incidence of POD when anesthesia was induced with ketamine (1–2 mg/kg) or propofol (0.5–1 mg/kg). For elderly patients undergoing emergency surgery (where the risk of POD is 1.5–3 times that of elective surgery), a single preoperative administration of ketamine (1 mg/kg) resulted in a lower crude incidence of delirium (10%) compared with placebo (75%) [29]. However, after adjusting for confounding factors, the risk of delirium instead increased by 3 times (odds ratio [OR] = 3.012, *p* = 0.013).

To summarize, ketamine may not increase the incidence of POD in pediatric patients. However, for elderly patients, there is significant heterogeneity in the results, and greater caution is required, especially in emergency surgery.

## 9. Esketamine

Esketamine is a derivative of ketamine, noted for its reduced cognitive side effects in comparison to its counterpart. Clinically, esketamine is frequently employed in the treatment of depression, and considering the strong correlation between depression and delirium, researchers hypothesized that it may possess a preventive effect against delirium.

Based on current RCTs, esketamine does not seem to increase the incidence of POD in pediatric patients. A prospective, triple-blind RCT was conducted on children aged 1–6 years scheduled for fiberoptic bronchoscopy. Researchers evaluated the effect of intranasal esketamine (1.0 mg/kg) versus intranasal dexmedetomidine (1.0 μg/kg) on ED using the Cornell Assessment of Pediatric Delirium (CAPD) scale. The results showed no statistically significant difference (7.14% vs. 18.60%, *p* > 0.05) [30]. Another trial also obtained similar results using intranasal drops of dexmedetomidine (2 μg/kg) combined with esketamine (2 mg/kg) [31]. Additionally, other researchers added low-dose esketamine to propofol administered intraoperatively [32]. The results showed that the POD scores did not increase, but the time to regain consciousness and the length of stay in the post-anesthesia care unit (PACU) were significantly prolonged.

In elderly patients, previous studies using the CAM scale for assessment found that using esketamine at a dose of 1 mg/kg for patient-controlled intravenous analgesia (PCIA) following gastrointestinal surgery could significantly reduce the incidence of POD compared with sufentanil (13.3% vs. 40%, *p* = 0.041) [33]. Researchers believe this may be due to the better postoperative analgesic effect and a decrease in interleukin-6 (IL-6) levels in patients in the esketamine group. However, other RCTs have reached different conclusions. Researchers used the 3-min Diagnostic Interview for Confusion Assessment Method (3D-CAM) to evaluate elderly patients over 60 years old who underwent total hip/knee arthroplasty and received low-dose esketamine (0.2 mg/kg for induction, 0.125 mg/kg/h for intraoperative maintenance, and 0.5 mg/kg for postoperative analgesia) [34]. The results showed that this regimen failed to reduce the incidence of POD within 3 days after surgery (8.5% in the esketamine group vs. 10.8% in the placebo group, *p* = 0.528), and there were no significant differences in the subtype or duration of delirium—despite the significant reduction in postoperative pain in the esketamine group. Additionally, other researchers administered a single dose of esketamine (0.2 mg/kg) to elderly patients undergoing elective non-cardiac surgery, and also found no reduction in the incidence of POD (assessed by 3D-CAM), with no significant difference in Numerical Rating Scale (NRS) scores either [35]. Current research indicates that there is significant heterogeneity in the preventive effect of esketamine on POD in elderly patients, and its efficacy is influenced by multiple factors such as dosage, surgical type, assessment method, and patients’ baseline characteristics. Future studies need to maximize the clinical benefits of esketamine through individualized dosage adjustment, precise surgical stratification, and multi-dimensional assessment.

Researchers conducted similar RCTs in non-elderly adult patients. In a randomized, triple-blind, controlled trial involving 116 patients undergoing cardiac valve surgery with cardiopulmonary bypass, anesthesia induction with esketamine versus placebo was compared [36]. The results showed that intravenous administration of esketamine (0.25 mg/kg) before anesthesia induction significantly reduced the incidence of POD in relatively younger American Society of Anesthesiologists II-III cardiac surgery patients (23.2% vs. 44.6%, RR = 0.52, *p* = 0.018). In another RCT involving breast cancer patients aged 18–55 years undergoing unilateral modified radical mastectomy, researchers administered a single intravenous dose of 0.2 mg/kg subanesthetic esketamine after anesthesia induction [37]. The results showed that esketamine did not increase the incidence of POD. In addition, several researchers discovered that esketamine did not result in any additional neuropsychiatric side effects, including POD, in patients with severe depression undergoing electroconvulsive therapy [38].

Based on these findings, the effect of esketamine on POD shows marked population dependence, as well as dose-dependent and surgery-specific characteristics. Its efficacy is also affected by the interaction of multiple factors. However, it is reassuring that there are currently no reports indicating that it increases the incidence of POD. Furthermore, exploring whether esketamine can improve the incidence of POD in patients with varying severities of depression undergoing non-depressive treatments or surgeries may be of certain research value.

## 10. Benzodiazepines

Benzodiazepines are among the most frequently used adjuncts in anesthesia, often deployed to prevent intraoperative awareness due to their amnesic properties. Traditionally, there has been a prevailing belief that benzodiazepines are linked to an increased risk of delirium. With recent advancements in anesthetic pharmacology, a novel short-acting benzodiazepine, remimazolam, may introduce a new paradigm in cognitive outcomes compared to its predecessors.

### 10.1. Midazolam

Midazolam is a classic short-acting benzodiazepine, renowned for its sedative, anxiolytic, and anticonvulsant properties. A multicenter study conducted across nine hospitals in Germany further corroborated that midazolam does not elevate the incidence of POD [39]. Moreover, research suggested that a dosage of 0.1 mg/kg of midazolam has a similar impact on POD as 2 μg/kg of intranasal dexmedetomidine [40]. In a comprehensive study on 19,768 patients across 20 cardiac surgery centers in North America, researchers compared restrictive versus liberal administration of benzodiazepines during anesthesia. The findings demonstrated that restricting benzodiazepines during cardiac procedures did not significantly reduce the incidence of POD. However, the authors suggested that, given the potential for smaller effect sizes, limiting benzodiazepine use might still be warranted [41]. Notably, the study did not clearly define the age limits of participants, although it was noted that the average age was 65, along with various age subgroup analyses. Conversely, in a comparative study, researchers contended that oral midazolam could effectively reduce the incidence and severity of EA, albeit not as effectively as dexmedetomidine [42]. Historically, midazolam has been one of the most extensively investigated drugs; however, recent research trends have shifted towards examining the implications of restricted use on POD. Current findings do not support the longstanding belief that midazolam exacerbates the risk of POD. However, as a precaution, we recommend restricting its use as much as possible in elderly patients and during cardiac surgery.

### 10.2. Remimazolam

Remimazolam is a recently developed ultra-short-acting benzodiazepine. Distinct from traditional benzodiazepines, remimazolam is primarily metabolized by tissue carboxylesterases. As a result, researchers have undertaken extensive investigations to explore its properties.

In a study on children undergoing laparoscopic inguinal hernia repair with sevoflurane anesthesia, it was found that both a single preoperative injection and continuous intraoperative infusion of remimazolam significantly diminished the incidence of POD [43]. In a cohort of children aged 3 to 6 years undergoing elective surgery, no increase in the incidence of ED was observed with the use of remimazolam for induction and maintenance of general anesthesia [44]. Remimazolam appears to be relatively safe for use in pediatric surgery. Although whether it can reduce POD remains uncertain, there is no evidence indicating that it increases the incidence of POD.

A prospective randomized controlled trial on 320 orthopedic elderly patients concluded that the administration of remimazolam was not linked to an increased incidence of POD [45]. Moreover, varying doses of remimazolam did not reveal a correlation between escalating dosages and heightened POD rates. Another investigation found that, when compared to propofol, remimazolam significantly reduced the incidence of EA in elderly patients undergoing hip replacement surgery [46]. However, this outcome was not replicated in patients undergoing radical resection for colon cancer, laparoscopic cholecystectomy, or transurethral bladder tumor resection [47]. In elderly patients receiving non-intubated general anesthesia, remimazolam exhibited comparable effects to dexmedetomidine concerning the incidence of EA [48]. Based on the above studies, we consider that remimazolam has not increased the incidence of POD in elderly patients either, which may be attributed to its unique pharmacokinetics.

Similarly, the research findings on the application of remimazolam at routine doses in other populations are also similar. One study revealed that in adult patients undergoing cerebrovascular surgery, the administration of remimazolam (0.1 mg/kg for induction and 0.3–0.7 mg/kg/h for maintenance) did not lead to an increased incidence of EA, and the modified Rankin Scale (mRS) scores at both 30 and 90 days postoperation remained stable [49]. In adults aged 18 to 65 undergoing nasal surgery, researchers posited that the administration of remimazolam at 1–2 mg/kg/h during the procedure, or a slow intravenous infusion of 0.1 mg/kg postoperatively, could effectively mitigate both the incidence and severity of EA [50].

Future inquiries could explore its applications in high-risk scenarios, such as in patients on long-term psychiatric medications, those with brain tumors, individuals undergoing cochlear implantation, or in the context of major cardiac surgeries.

## 11. Opioids

As a vital component of balanced anesthesia, opioids demonstrate remarkable analgesic efficacy. Their incorporation has substantially diminished the necessity for other anesthetic agents, thereby alleviating many adverse events associated with those agents. Historically, opioids have been thought to have a close correlation with the incidence of perioperative delirium. However, recent meta-analyses presented a contrasting perspective that perioperative administration of opioids showed no significant association with POD.

Opioids can be classified based on their interaction with opioid receptors into agonists, partial agonists, agonist-antagonists, and antagonists. Researchers conducted clinical trials on a variety of opioid medications. In a study on children undergoing outpatient dental procedures, the administration of alfentanil at doses of 0.2 μg/kg/min or 0.4 μg/kg/min, followed by assessment with the PAED scale, demonstrated a significant reduction in the incidence of ED [51]. Additionally, intravenous administration of fentanyl at different time points (10–15 min before the conclusion of surgery versus at the end of surgery) showed no significant impact on the occurrence of POD [52]. Some studies showed that the efficacy of remifentanil in reducing POD is dose-dependent; however, this effect is accompanied by an increase in other adverse events [53]. Furthermore, research suggested that continuous application of remifentanil during the awakening phase may also mitigate the incidence of ED [54]. A comparative investigation contrasting low-dose intrathecal morphine analgesia with PCIA using sufentanil revealed that morphine administration significantly lowered the incidence of POD in elderly patients undergoing hip fracture surgery [55]. Another study indicated that a combination of serratus anterior plane block with 0.1 mg/kg of oxycodone was more effective in reducing POD than 0.1 μg/kg of sufentanil [56]. The mixed agonist-antagonist nalbuphine has also been shown to diminish the incidence of ED in children following adenotonsillectomy [57]. In cochlear implantation procedures, which exhibit an ED incidence as high as 80% in children, a dose of 0.2 mg/kg of nalbuphine was found to be particularly effective [58].

In general, current studies have shown that alfentanil at 0.2–0.4 μg/kg/min, remifentanil at 0.25 μg/kg/min, nalbuphine at 0.2 mg/kg (when used intraoperatively), as well as low-dose intrathecal morphine and oxycodone at 0.1 mg/kg (when used as adjuvants to nerve block), exert a certain preventive effect on POD. These drugs can serve as adjuvants to opioid receptor agonists, the most commonly used drugs in general anesthesia.

## 12. Local Anesthetics

Lidocaine can also provide anesthetic effects through intravenous administration. One study revealed that in elderly patients undergoing hip fracture surgery, an infusion of lidocaine at a rate of 1.5 mg/kg/h significantly decreased both the incidence and severity of POD [59]. Furthermore, when lidocaine was employed as a substitute for opioids in intravenous anesthesia, researchers found no significant difference in the occurrence of POD between the two groups, while hemodynamic stability was markedly enhanced [60].

## 13. Dexmedetomidine

Dexmedetomidine is a highly selective α2 adrenergic receptor agonist that induces sedative and hypnotic effects by acting on presynaptic and postsynaptic α2 receptors in the locus coeruleus, thereby stimulating endogenous sleep pathways [61]. Notably, it achieves sedation without significant respiratory depression or other adverse effects. Although dexmedetomidine is not strictly classified as a general anesthetic, it is an essential medication for clinical anesthesia. Therefore, this review classifies it together with anesthetic agents for discussion. Additionally, as many as 40 RCTs on dexmedetomidine were included, but individual references have been omitted due to space constraints.

In recent years, researchers have conducted extensive research on the effect of dexmedetomidine on POD, covering populations ranging from children to the elderly and surgical types from outpatient surgeries to cardiac surgeries. Overall, dexmedetomidine shows significant potential in preventing POD, but its efficacy varies depending on the patient population, surgical type, administration timing, dosage, and route (Table 2). In pediatric patients, dexmedetomidine has a potential role in reducing POD. For elderly patients, it shows a tendency to reduce the incidence of POD in major non-cardiac surgery, but no significant effect has been observed in minimally invasive surgery. However, its effect on POD remains unclear in cardiac surgery in elderly patients. For other adult patients, dexmedetomidine does not show an effective role in reducing POD in cardiac surgery, but it can effectively reduce POD in non-cardiac surgery. Overall, dexmedetomidine has shown potential in preventing POD, but its efficacy varies by patient population, surgical type, administration timing, dosage, and route.

## 14. Limitations

Although progress has been made in current research on general anesthetics and POD, there are still limitations across multiple dimensions.

First, in terms of study design, most RCTs are single-center studies with generally small sample sizes. Significant differences exist between studies in aspects such as anesthetic regimens, drug dosages, infusion rates, and postoperative management, making it difficult to directly compare the results. In clinical practice, anesthesia often adopts multidrug combination regimens; however, existing studies mostly focus on the independent effects of a single drug and lack dose–response relationship analysis.

Second, regarding POD diagnosis and monitoring, there are significant differences in POD diagnostic criteria used in different studies. Differences in sensitivity and specificity between assessment tools result in low comparability of study results. Most studies only monitor the incidence of POD 3–7 days postoperatively, yet POD may recur weeks or even months after surgery. POD is characterized by fluctuations and requires multiple daily assessments to capture peak episodes. However, in actual research, only 28% of RCTs follow the guideline recommendation of “at least twice daily assessments from postoperative day 1 to day 5.”

Finally, in terms of confounding factor control, some studies fail to fully control confounding factors that may affect POD. For example, in studies comparing volatile anesthetics and propofol in cardiac surgery, additional propofol use during patients’ ICU stay was not restricted; some studies on elderly patients did not exclude the interference of postoperative dexmedetomidine or analgesic drugs on POD assessment; other studies did not adjust for factors such as intraoperative hypotension and postoperative infection, leading to resultant bias and inability to accurately reflect the true association between anesthetics and POD.

## 15. Conclusions

Currently, no single general anesthetic has been proven to universally and significantly alter the risk of POD; the effect of these anesthetics on modifying POD risk is highly dependent on patient-specific factors, surgical types, and specific details of perioperative management. Dexmedetomidine has shown preventive potential in various scenarios; novel drugs (remimazolam, ciprofol, esketamine) also do not appear to increase the risk of POD, providing new options for personalized anesthesia. Meanwhile, the risks of certain drugs in traditional perceptions may have been overestimated. In addition, this review mainly focuses on the prevention and treatment of POD; anesthesiologists should also consider comprehensive factors such as hemodynamics and tolerance when using these drugs in clinical practice. To address current knowledge gaps, future research should prioritize the following: (1) Large-scale, multicenter randomized controlled trials with standardized protocols for anesthetic administration, POD assessment (including twice-daily evaluations from postoperative day 1 to 5), and adequate control of confounding factors (e.g., perioperative opioid and dexmedetomidine use); (2) Stratified analyses by surgical type (cardiac vs. non-cardiac, elective vs. emergency) and patient subpopulations (elderly, pediatric, and those with baseline cognitive impairment or depression); (3) Investigation of dose–response relationships and combination regimens (e.g., propofol–ketamine, dexmedetomidine–opioid) rather than single-agent effects alone; and (4) Longer follow-up periods to capture late or recurrent delirium episodes. The core of future research lies in optimizing drug selection and multimodal analgesia regimens for specific high-risk populations and surgical types through precision medicine strategies, thereby achieving effective prevention and management of POD.

## Figures and Tables

**Table 1:** GRADE-Based Recommendations for General Anesthetics on Postoperative Delirium by Population and Surgery Type.

Population	Surgery Type	Drug	Suggestion	Evidence Quality *
Children (<18 y)	Non-cardiac	Propofol	Recommended	Low
		Ciprofol	Insufficient Evidence	Low
		Sevoflurane	Use with Caution	Low
		Ketamine	Use with Caution	Low
		Esketamine	Use with Caution	Low
		Midazolam	Use with Caution	Moderate
		Remimazolam	Use with Caution	Moderate
		Opioids	Recommendation	Low
		Dexmedetomidine	Recommendation	High
Elderly (≥65 y)	Non-cardiac	Ciprofol	Insufficient Evidence	Low
		Sevoflurane	Use with Caution	Low
		Ketamine	Use with Caution	Very low
		Esketamine	Use with Caution	Low
		Midazolam	Use with Caution	Moderate
		Remimazolam	Recommended	Moderate
		Opioids	Intrathecal Administration Recommended	Low
		Lidocaine	Recommended	Low
		Dexmedetomidine	Recommended	Moderate
	Cardiac	Sevoflurane	Use with Caution	Low
		Ketamine	Insufficient evidence	Low
		Midazolam	Use with Caution	Moderate
		Dexmedetomidine	Not Recommended	High
Adults (18–64 y)	Cardiac	Sevoflurane	Use with Caution	Low
		Esketamine	Recommended	Low
		Dexmedetomidine	Not Recommended	Low
	Non-cardiac	Sevoflurane	Use with Caution	Low
		Esketamine	Use with Caution	Low
		Remimazolam	Recommended	Moderate
		Dexmedetomidine	Recommended	Moderate

* Notes: Employ GRADE (Grading of Recommendations Assessment, Development and Evaluation) for grading the levels of evidence.

**Table 2:** Effects of dexmedetomidine on POD in different populations.

Population	Type of Surgery	RCTs (Cases)	Sample Size (Cases)	Decrease	No Difference	Quality of Evidence *
Pediatric	Non-cardiac surgery	8	1021	7	1	High
Elderly	Non-cardiac surgery	19	1612	14	5	Moderate
	Cardiac surgery	4	866	1	3	High
Adults	Non-cardiac surgery	4	1842	3	1	Moderate
	Cardiac surgery	5	502	1	4	Low

* Notes: Employ GRADE (Grading of Recommendations Assessment, Development and Evaluation) for grading the levels of evidence.

## Data Availability

Not applicable.

## Abbreviations

The following abbreviations are used in this manuscript:

POD	Postoperative delirium
RCTs	Randomized controlled trials
ED	Emergence delirium
EA	Emergence agitation
GRADE	Grading of Recommendations Assessment, Development and Evaluation
GABAA	Gamma-Aminobutyric Acid Type A
PAED	Pediatric Anesthesia Emergence Delirium Scale
RR	Risk Ratio
CI	Confidence Interval
RD	Risk Difference
CAM	Confusion Assessment Method
EEG	Electroencephalogram
ICDSC	Intensive Care Delirium Screening Checklist
AUC	Area Under the Curve
IA	Inhalational Anesthesia
TIVA	Total Intravenous Anesthesia
CAM-ICU	Confusion Assessment Method for the Intensive Care Unit
NMDA	N-methyl-D-aspartate
CABG	Coronary Artery Bypass Grafting
CAPD	Cornell Assessment of Pediatric Delirium
PACU	Post-Anesthesia Care Unit
PCIA	Patient-Controlled Intravenous Analgesia
IL-6	Interleukin-6
3D-CAM	3-Min Diagnostic Interview for Confusion Assessment Method
NRS	Numerical Rating Scale
mRS	Modified Rankin Scale

## References

[1] Wilson J.E., Mart M.F., Cunningham C., Shehabi Y., Girard T.D., MacLullich A.M.J., Slooter A.J.C., Ely E.W. (2020). Delirium. Nat. Rev. Dis. Primers.

[2] Liu X., Huangfu Z., Zhang X., Ma T. (2025). Global Research Trends in Postoperative Delirium and Its Risk Factors: A Bibliometric and Visual Analysis. J. Perianesth Nurs..

[3] Vreeswijk R., Maier A.B., Kalisvaart K.J. (2022). Recipe for primary prevention of delirium in hospitalized older patients. Aging Clin. Exp. Res..

[4] Weiser T.G., Regenbogen S.E., Thompson K.D., Haynes A.B., Lipsitz S.R., Berry W.R., Gawande A.A. (2008). An estimation of the global volume of surgery: A modelling strategy based on available data. Lancet.

[5] Subramaniyan S., Terrando N. (2019). Neuroinflammation and Perioperative Neurocognitive Disorders. Anesth. Analg..

[6] Hshieh T.T., Fong T.G., Marcantonio E.R., Inouye S.K. (2008). Cholinergic deficiency hypothesis in delirium: A synthesis of current evidence. J. Gerontol. A Biol. Sci. Med. Sci..

[7] Palanca B.J.A., Wildes T.S., Ju Y.S., Ching S., Avidan M.S. (2017). Electroencephalography and delirium in the postoperative period. Br. J. Anaesth..

[8] Ni P., Dong H., Wang Y., Zhou Q., Xu M., Qian Y., Sun J. (2018). IL-17A contributes to perioperative neurocognitive disorders through blood-brain barrier disruption in aged mice. J. Neuroinflamm..

[9] Su X., Meng Z.T., Wu X.H., Cui F., Li H.L., Wang D.X., Zhu X., Zhu S.N., Maze M., Ma D. (2016). Dexmedetomidine for prevention of delirium in elderly patients after non-cardiac surgery: A randomised, double-blind, placebo-controlled trial. Lancet.

[10] Inouye S.K., Westendorp R.G., Saczynski J.S. (2014). Delirium in elderly people. Lancet.

[11] Chen J., Shi X., Hu W., Lin R., Meng L., Liang C., Ma X., Xu L. (2025). Comparing different administration methods of subanaesthetic propofol to mitigate emergence agitation in preschool children undergoing day surgery: A double-blind, randomised controlled study. BMJ Paediatr. Open.

[12] Zhou J., Wang L., Zhong Z., Yuan L., Huang J., Zou P., Cao X., Peng D., Liao B., Zeng J. (2025). Pharmacological mechanism and clinical application of ciprofol. Front. Pharmacol..

[13] Zhang S., Liu Y., Liu Y., Xu T. (2025). The Recovery of Ciprofol, Remimazolam and Remimazolam-Flumazenil for General Anesthesia Undergoing Fundus Surgery: A Single-Center, Prospective, Randomized, Controlled Clinical Study. Drug Des. Devel Ther..

[14] McGuigan S., Evered L., Scott D.A., Silbert B., Zetterberg H., Blennow K. (2022). Comparing the effect of xenon and sevoflurane anesthesia on postoperative neural injury biomarkers: A randomized controlled trial. Med. Gas. Res..

[15] Mori K., Komatsu T., Fujiwara Y., Fujita Y. (2024). Comparison of the Effects of Desflurane and Sevoflurane on Variations in Salivary Melatonin and Sleep Disturbance After Total Knee Arthroplasty: A Single-center, Prospective, Randomized, Controlled, Open-label Study. J. Perianesth Nurs..

[16] Kim Y.S., Kim J., Park S., Kim K.N., Ha Y., Yi S., Shin D.A., Kuh S.U., Lee C.K., Koo B.N. (2024). Differential effects of sevoflurane and desflurane on frontal intraoperative electroencephalogram dynamics associated with postoperative delirium. J. Clin. Anesth..

[17] Peker K., Polat R. (2020). Effects of intravenous and mask induction on post-operative emergence delirium in pediatric patients undergoing tonsillectomy with or without adenoidectomy. Ir. J. Med. Sci..

[18] Quintão V.C., Carlos R.V., Cardoso P.F.N., Zeferino S.P., Kulikowski L.D., Lee-Archer P., Carmona M.J.C. (2023). Comparison of intravenous and inhalation anesthesia on postoperative behavior changes in children undergoing ambulatory endoscopic procedures: A randomized clinical trial. Paediatr. Anaesth..

[19] Farrer T.J., Monk T.G., McDonagh D.L., Martin G., Pieper C.F., Koltai D. (2025). A prospective randomized study examining the impact of intravenous versus inhalational anesthesia on postoperative cognitive decline and delirium. Appl. Neuropsychol. Adult.

[20] Shin S., Kim S.H., Park K.K., Kim S.J., Bae J.C., Choi Y.S. (2020). Effects of Anesthesia Techniques on Outcomes after Hip Fracture Surgery in Elderly Patients: A Prospective, Randomized, Controlled Trial. J. Clin. Med..

[21] Cao S.J., Zhang Y., Zhang Y.X., Zhao W., Pan L.H., Sun X.D., Jia Z., Ouyang W., Ye Q.S., Zhang F.X. (2023). Delirium in older patients given propofol or sevoflurane anaesthesia for major cancer surgery: A multicentre randomised trial. Br. J. Anaesth..

[22] Duan G.Y., Duan Z.X., Chen H., Chen F., Chen F., Du Z.Y., Chen L.Y., Lu K.Z., Zuo Z.Y., Li H. (2023). Cognitive function and delirium following sevoflurane or propofol anesthesia for valve replacement surgery: A multicenter randomized controlled trial. Kaohsiung J. Med. Sci..

[23] Varsha A.V., Unnikrishnan K.P., Saravana Babu M.S., Raman S.P., Koshy T. (2024). Comparison of Propofol-Based Total Intravenous Anesthesia versus Volatile Anesthesia with Sevoflurane for Postoperative Delirium in Adult Coronary Artery Bypass Grafting Surgery: A Prospective Randomized Single-Blinded Study. J. Cardiothorac. Vasc. Anesth..

[24] Qian B., Zheng W., Shi J., Chen Z., Guo Y., Yao Y. (2020). Ketamine Enhances Intranasal Dexmedetomidine-Induced Sedation in Children: A Randomized, Double-Blind Trial. Drug Des. Devel Ther..

[25] Biricik E., Karacaer F., Tunay D.L., Ilgınel M., Küçükbingöz Ç. (2024). The Effect of Different Propofol-Ketamine Combinations on Emergence Delirium in Children Undergoing Adenoidectomy and Tonsillectomy Surgery. J. Perianesth Nurs..

[26] Abd Ellatif S.E., Mowafy S.M.S., Shahin M.A. (2024). Ketofol versus Dexmedetomidine for preventing postoperative delirium in elderly patients undergoing intestinal obstruction surgeries: A randomized controlled study. BMC Anesthesiol..

[27] Siripoonyothai S., Sindhvananda W. (2021). Comparison of postoperative delirium within 24 hours between ketamine and propofol infusion during cardiopulmonary bypass machine: A randomized controlled trial. Ann. Card. Anaesth..

[28] Wittwer E.D., Cerhan J.H., Schroeder D.R., Schaff H.V., Mauermann W.J. (2023). Impact of ketamine versus propofol for anesthetic induction on cognitive dysfunction, delirium, and acute kidney injury following cardiac surgery in elderly, high-risk patients. Ann. Card. Anaesth..

[29] Ghazaly H.F., Hemaida T.S., Zaher Z.Z., Elkhodary O.M., Hammad S.S. (2023). A pre-anesthetic bolus of ketamine versus dexmedetomidine for prevention of postoperative delirium in elderly patients undergoing emergency surgery: A randomized, double-blinded, placebo-controlled study. BMC Anesthesiol..

[30] Xie W., Wang L., Peng Z., Zhang R., Dong Z.F., Huang Y., Wan Z.H., Wang L. (2025). The Impact of Preoperative Low-Dose Esketamine and Dexmedetomidine Nasal Administration on the Emergence Delirium in Children Undergoing Fiber Bronchoscopy: A Prospective Randomized Controlled Trial. Clin. Neuropharmacol..

[31] Zhang K., Zhang G., Zhang Y., Wang J., Bai J., Zheng J., Tao Y. (2025). Efficacy of intranasal dexmedetomidine-esketamine sedation for pediatric acceptance of facemask: Single-center, double-blind, randomized, controlled trial. BMC Anesthesiol..

[32] Han D., Du X., Li Y., Wang Y., Wei L., Zhang L., Li F., Pan S. (2024). Supplemental low-dose esketamine to propofol versus propofol alone on perioperative characteristics in children undergoing surgery: A prospective randomized controlled trial. Minerva Anestesiol..

[33] Liu J., Wang T., Song J., Cao L. (2024). Effect of esketamine on postoperative analgesia and postoperative delirium in elderly patients undergoing gastrointestinal surgery. BMC Anesthesiol..

[34] Ma C.B., Zhang C.Y., Gou C.L., Liang Z.H., Zhang J.X., Xing F., Yuan J.J., Wei X., Zhang Y.B., Wang Z.Y. (2024). Effect of Low-Dose Esketamine on Postoperative Delirium in Elderly Patients Undergoing Total Hip or Knee Arthroplasty: A Randomized Controlled Trial. Drug Des. Devel Ther..

[35] Zhang Y., Chen R., Tang S., Sun T., Yu Y., Shi R., Wang K., Zeng Z., Liu X., Meng Q. (2024). Diurnal variation of postoperative delirium in elderly patients undergoing esketamine anesthesia for elective noncardiac surgery: A randomized clinical trial. Int. J. Surg..

[36] Xiong X., Shao Y., Chen D., Chen B., Lan X., Shi J. (2024). Effect of Esketamine on Postoperative Delirium in Patients Undergoing Cardiac Valve Replacement with Cardiopulmonary Bypass: A Randomized Controlled Trial. Anesth. Analg..

[37] Wang H., Te R., Zhang J., Su Y., Zhou H., Guo N., Chi D., Huang W. (2024). Effects of a single subanesthetic dose of esketamine on postoperative subthreshold depressive symptoms in patients undergoing unilateral modified radical mastectomy: A randomised, controlled, double-blind trial. BMC Psychiatry.

[38] Ren L., Chen Q., Gao J., Liu Y., Tao Y., Li X., Luo Q., Lv F., Min S. (2024). Clinical efficacy of adjunctive esketamine anesthesia in electroconvulsive therapy for major depressive disorders: A pragmatic, randomized, controlled trial. Psychiatry Res..

[39] Kowark A., Keszei A.P., Schneider G., Pilge S., Schneider F., Obert D.P., Georgii M.T., Heim M., Rossaint R., Ziemann S. (2024). Preoperative Midazolam and Patient-Centered Outcomes of Older Patients: The I-PROMOTE Randomized Clinical Trial. JAMA Surg..

[40] Shen F., Zhang Q., Xu Y., Wang X., Xia J., Chen C., Liu H., Zhang Y. (2022). Effect of Intranasal Dexmedetomidine or Midazolam for Premedication on the Occurrence of Respiratory Adverse Events in Children Undergoing Tonsillectomy and Adenoidectomy: A Randomized Clinical Trial. JAMA Netw. Open.

[41] Spence J., Devereaux P.J., Lee S.F., D’Aragon F., Avidan M.S., Whitlock R.P., Mazer C.D., Rousseau-Saine N., Rajamohan R.R., Pryor K.O. (2025). Benzodiazepine-Free Cardiac Anesthesia for Reduction of Postoperative Delirium: A Cluster Randomized Crossover Trial. JAMA Surg..

[42] Algyar M.F., Abdelghany A.M., Arafa S.K., Elsayed A.A. (2025). A Placebo-Controlled Randomized Trial Comparing Oral Midazolam, Dexmedetomidine, and Gabapentin on Prophylaxis of Emergence Agitation After Sevoflurane Anesthesia in Adenotonsillectomy. Pain. Physician.

[43] Cai Y.H., Zhong J.W., Ma H.Y., Szmuk P., Wang C.Y., Wang Z., Zhang X.L., Dong L.Q., Liu H.C. (2024). Effect of Remimazolam on Emergence Delirium in Children Undergoing Laparoscopic Surgery: A Double-blinded Randomized Trial. Anesthesiology.

[44] Fang Y.B., Zhong J.W., Szmuk P., Lyu Y.L., Xu Y., Qu S., Du Z., Shangguan W., Liu H.C. (2025). Safety and efficacy of remimazolam tosilate for general anaesthesia in paediatric patients undergoing elective surgery: A multicentre, randomised, single-blind, controlled trial. Anaesthesia.

[45] Yang J.J., Lei L., Qiu D., Chen S., Xing L.K., Zhao J.W., Mao Y.Y., Yang J.J. (2023). Effect of Remimazolam on Postoperative Delirium in Older Adult Patients Undergoing Orthopedic Surgery: A Prospective Randomized Controlled Clinical Trial. Drug Des. Devel Ther..

[46] Duan J., Ju X., Wang X., Liu N., Xu S., Wang S. (2023). Effects of Remimazolam and Propofol on Emergence Agitation in Elderly Patients Undergoing Hip Replacement: A Clinical, Randomized, Controlled Study. Drug Des. Devel Ther..

[47] Jeon Y.G., Kim S., Park J.H., Lee J., Song S.A., Lim H.K., Song S.W. (2023). Incidence of intraoperative hypotension in older patients undergoing total intravenous anesthesia by remimazolam versus propofol: A randomized controlled trial. Medicine.

[48] Deng Y., Qin Z., Wu Q., Liu L., Yang X., Ju X., Zhang Y., Liu L. (2022). Efficacy and Safety of Remimazolam Besylate versus Dexmedetomidine for Sedation in Non-Intubated Older Patients with Agitated Delirium After Orthopedic Surgery: A Randomized Controlled Trial. Drug Des. Devel Ther..

[49] Zhang J., Zhang J., Wang Y., Bai X., Guo Q., Liu W., Li H., Zhu F., Wang X., Jiang X. (2024). Effect of remimazolam vs propofol on emergence from general anesthesia in patients undergoing cerebral endovascular procedures: A randomized controlled, non-inferiority trial. J. Clin. Anesth..

[50] Cho S.A., Ahn S.M., Kwon W., Sung T.Y. (2024). Comparison of remimazolam and desflurane in emergence agitation after general anesthesia for nasal surgery: A prospective randomized controlled study. Korean J. Anesthesiol..

[51] Zhao N., Zeng J., Fan L., Zhang C., Wu Y., Wang X., Gao F., Yu C. (2022). The Effect of Alfentanil on Emergence Delirium Following General Anesthesia in Children: A Randomized Clinical Trial. Paediatr. Drugs.

[52] Kim H.J., Kim M.S., Kim H.Y., Park W.K., Kim W.S., Kim S., Kim H.J. (2020). Effect of Timing of Intravenous Fentanyl Administration on the Incidence of Posttonsillectomy Nausea and Vomiting. Laryngoscope.

[53] Ma J., Wang Y., Liu Z., Han S. (2025). Effects of different doses of remifentanil combined with sevoflurane anesthesia on postoperative analgesia and hemodynamics in pediatric patients undergoing laparoscopic inguinal hernia repair. BMC Anesthesiol..

[54] Choi E.K., Lee S., Kim W.J., Park S.J. (2018). Effects of remifentanil maintenance during recovery on emergence delirium in children with sevoflurane anesthesia. Paediatr. Anaesth..

[55] Xu Z., Tang Z., Yao J., Liang D., Jin F., Liu Y., Guo K., Yang X. (2022). Comparison of low-dose morphine intrathecal analgesia and sufentanil PCIA in elderly patients with hip fracture undergoing single spinal anesthesia—A randomized clinical trial. BMC Anesthesiol..

[56] Wang T., Wang Q.B., Hou Z.J., Chen W., Cheng H., He J.K., Zhu L.L., Wang Y.L., Chen Y.Q. (2024). Effect of serratus anterior plane block combined with oxycodone for transition analgesia on preventing emergence agitation after video-assisted thoracoscopic surgery: A randomized controlled trial. Sci. Rep..

[57] He J., Zhang L., Tao T., Wen X., Chen D., Zheng X., Luo C., Liang H., Wang H. (2023). Nalbuphine reduces the incidence of emergence agitation in children undergoing Adenotonsillectomy: A prospective, randomized, double-blind, multicenter study. J. Clin. Anesth..

[58] Li Y., Li Q., Zhao G., Zhang H., Zhong H., Zeng Y. (2024). Nalbuphine in Pediatric Emergence Agitation Following Cochlear Implantation: A Randomized Trial. Drug Des. Devel Ther..

[59] Li X., Wu J., Lan H., Shan W., Xu Q., Dong X., Duan G. (2023). Effect of Intraoperative Intravenous Lidocaine on Postoperative Delirium in Elderly Patients with Hip Fracture: A Prospective Randomized Controlled Trial. Drug Des. Devel Ther..

[60] Amin S.M., Hasanin A., ElSayed O.S., Mostafa M., Khaled D., Arafa A.S., Hassan A. (2023). Comparison of the hemodynamic effects of opioid-based versus lidocaine-based induction of anesthesia with propofol in older adults: A randomized controlled trial. Anaesth. Crit. Care Pain. Med..

[61] He X., Cheng K.M., Duan Y.Q., Xu S.S., Gao H.R., Miao M.Y., Li H.L., Chen K., Yang Y.L., Zhang L. (2021). Feasibility of low-dose dexmedetomidine for prevention of postoperative delirium after intracranial operations: A pilot randomized controlled trial. BMC Neurol..

